# Nociception in the Glycine Receptor Deficient Mutant Mouse Spastic

**DOI:** 10.3389/fnmol.2022.832490

**Published:** 2022-04-25

**Authors:** Teja Wolfgang Groemer, Antoine Triller, Hanns Ulrich Zeilhofer, Kristina Becker, Volker Eulenburg, Cord Michael Becker

**Affiliations:** ^1^Institut für Biochemie, Emil-Fischer-Zentrum, Friedrich-Alexander-Universität Erlangen-Nürnberg, Erlangen, Germany; ^2^École Normale Supérieure, INSERM U 497 Biologie Cellulaire de la Synapse Normale et Pathologique, Paris, France; ^3^Institut für Experimentelle und Klinische Pharmakologie und Toxikologie, Emil-Fischer-Zentrum, Universität Erlangen-Nürnberg, Erlangen, Germany; ^4^Department für Anästhesiologie und Intensivmedizin, Universität Leipzig, Leipzig, Germany

**Keywords:** glycine receptor, pain, glycinergic inhibition, nociception, synaptic clustering, spastic

## Abstract

Glycine receptors (GlyRs) are the primary mediators of fast inhibitory transmission in the mammalian spinal cord, where they modulate sensory and motor signaling. Mutations in GlyR genes as well as some other genes underlie the hereditary disorder hyperekplexia, characterized by episodic muscle stiffness and exaggerated startle responses. Here, we have investigated pain-related behavior and GlyR expression in the spinal cord of the GlyR deficient mutant mouse *spastic* (*spa*). In *spastic* mice, the GlyR number is reduced due to a β subunit gene (*Glrb*) mutation resulting in aberrant splicing of GlyRβ transcripts. *Via* direct physical interaction with the GlyR anchoring protein gephyrin, this subunit is crucially involved in the postsynaptic clustering of heteromeric GlyRs. We show that the mutation differentially affects aspects of the pain-related behavior of homozygous Glrb^spa^/Glrb^spa^ mice. While response latencies to noxious heat were unchanged, chemically induced pain-related behavior revealed a reduction of the licking time and an increase in flinching in spastic homozygotes during both phases of the formalin test. Mechanically induced nocifensive behavior was reduced in spastic mice, although hind paw inflammation (by zymosan) resulted in allodynia comparable to wild-type mice. Immunohistochemical staining of the spinal cord revealed a massive reduction of dotted GlyRα subunit immunoreactivity in both ventral and dorsal horns, suggesting a reduction of clustered receptors at synaptic sites. Transcripts for all GlyRα subunit variants, however, were not reduced throughout the dorsal horn of *spastic* mice. These findings suggest that the loss of functional GlyRβ subunits and hence synaptically localized GlyRs compromises sensory processing differentially, depending on stimulus modality.

## Introduction

Strychnine-sensitive glycine receptors (GlyRs) are ligand-gated chloride channels mediating fast inhibitory transmission in the spinal cord and brainstem (Breitinger and Breitinger, 2020; Zeilhofer et al., 2021). These pentameric proteins are composed of α and β subunits. Although there is evidence for the existence of GlyRα homomeric receptors, α/β hetero-oligomers prevail in the ventral spinal cord during adulthood (Becker et al., 1988; Langosch et al., 1988). The subunit composition, however, is still controversially discussed ranging between a 2α3β stoichiometry to a 4α1β (Grudzinska et al., 2005; Yu et al., 2021). To date, four α subunits (α1–4) and one β subunit are known, all of which are expressed in the rodent spinal cord, but only the α subunits α1- α3 and the β subunit are being expressed in humans (Dutertre et al., 2012). Different GlyR subunits can be detected in the spinal cord dorsal horn where they exhibit a defined regional expression pattern in adult animals (Puskár et al., 2001; Harvey et al., 2004; Zeilhofer, 2005).

Spinal GlyRs serve functions in motor and sensory pathways: among others they mediate in the ventral spinal cord the recurrent inhibition of motoneurons conveyed by glycinergic Renshaw—interneurons (Gonzalez-Forero et al., 2005). In the dorsal part of the spinal cord, GlyRs dependent neurotransmission was shown to provide inhibitory input onto the projection neurons in the superficial dorsal horn (sfdh), thereby contributing to the discrimination of painful stimuli from other sensory input (Zeilhofer, 2005). Accordingly, allodynia can be induced by the intrathecal application of strychnine (Yaksh, 1989).

The function of glycine dependent neurotransmission on motor function has been extensively studied on mice carrying spontaneous function impairing mutation within the GlyR genes, Glra1 and Glrb that show phenotypes characterized by tremor, myoclonic episodes (startle), and a disturbed righting response (Becker et al., 1992; Buckwalter et al., 1994; Kingsmore et al., 1994; Mülhardt et al., 1994; Saul et al., 1994). Consistent with these data obtained in animals, mutations of the α1 or β glycine receptor subunits have been identified as causal for the development of the paroxysomal neurological disorder hyperekplexia in humans (Shiang et al., 1993; Chung et al., 2013). The murine phenotypes thereby resemble the human disorder (Becker et al., 2000). With the already demonstrated function of glycinergic neurotransmission in the processing of noxious stimuli, changes in pain perception have been demonstrated in human hyperekplexia patients (Vuilleumier et al., 2018). These findings, however, have not been recapitulated in the respective animal models in part due to the strong motor phenotype of the respective mouse models.

In contrast, genetic analysis of mice deficient in expression of the GlyR subunits GlyRα3 and α2 did not show any motor phenotype, demonstrating the heterogeneity of GlyR function in spinal signal processing. The GlyRα3 subunit in the sfdh was shown to contribute to the process of sensitization to inflammatory pain *via* a PGE2 receptor-induced phosphorylation of GlyRα3 containing receptors (Ahmadi et al., 2002; Harvey et al., 2004; Zeilhofer et al., 2021).

In this study, we have investigated nociception in homozygous spastic mice, that show a reduction in the amount of correctly processed protein and a concomitant decrease in the number of functional GlyRs in the plasma membrane (Becker et al., 1986, 1992) due to an insertion of a LINE-1 insertion in intron 5 of the *Glrb* gene (Kingsmore et al., 1994; Mülhardt et al., 1994), which leads to aberrant splicing of β-transcripts (Becker et al., 2012). Consistently, evoked glycinergic currents in α-motoneurons in the ventral horn are reduced in *spastic* mice (von Wegerer et al., 2003) and glycinergic inhibition is disrupted in the sfdh (Graham et al., 2003). To date, behavioral studies have focused on the startle and motor phenotype of the mice (Koch et al., 1996; Simon, 1997). Here, we have addressed the question of whether the electrophysiologically verified changes of glycinergic inhibition, result in alterations in pain-related behavior. Our results indicate that the disturbed inhibitory neurotransmission caused by Glrb gene mutation does not only results in the myoclonic startle reaction but differentially affects the behavioral response to noxious stimuli.

## Material and Methods

### Animals

Animals were maintained on a 12/12 h light dark cycle and had access to food and water *ad libitum*. All tests were performed in 6–10 weeks old spastic mice of hybrid genetic background [homozygous offspring of a C57Bl/6J Glrb^+/spa^ × F1 (C57Bl/6J Glrb^+/spa^ × C3H/HeJGlrb^+/+^)]. Wild-type littermates served as controls and animals of both sexes were used. Animals that underwent behavioral testing were not utilized for transcript analysis or immunohistochemistry. After completion of testing, mice were sacrificed by CO_2_ inhalation. All experiments were performed in accordance with the institutional guidelines of the University of Erlangen-Nürnberg and of the European Communities Council Directive (86/609/EEC).

### Transcript Analysis

Adult littermates were anesthetized with CO_2_, decapitated, and tissue was prepared in ice-cold PBS under a light microscope and directly transferred into Peq-Gold RNA-Pure^TM^ (Peq-Labs, Erlangen, Germany) for RNA extraction (*n* = 2 for each genotype). RNA was then isolated by phenol/chloroform extraction and transcribed into cDNA by superscript II reverse transcriptase (Invitrogen, Karlsruhe, Germany). PCR-amplification was performed with PAN-Script DNA polymerase (PAN Biotech, Aidenbach, Germany) in a Biometra T-Gradient (Biometra, Göttingen, Germany) thermocycler with 30–40 cycles of 94°C, 30 s; T annealing, 30 s, 72°C, 90 s. The following primers were used for PCR (5’–3’; forward, reverse): *Glra1*: CAACAGTTTCGGTTCCATC, CGCCTCTTCCTCCTAAATCGAAGCAGT; *Glra2*: GGGACAAACCACTTCAGGAGGC, TAGCATCTGCATCTTTGGGGGGT; *Glra3*: GATTTTACTTCTGGGAAGCCGC, GAACCACACCATCCTTTGCTTG, *Glra4*: CCCAATTTCAAAGTCCACCTGTG, CAATACCCTAGATCCTTCTCATCC; *Glrb*: GGATCCATTCAAGAGACA, AGCCACACATCCAGTGCCTT; *Actb*: TGAGACCTTCAACACCCCAG, CATCTGCTGGAAGGTGGACA.

### Immunohistochemistry

For immunostaining, mice of either sex were deeply anesthetized with pentobarbital (100 mg/kg) and perfused through the left ventricle with 4% (w/v) ice-cold formaldehyde in PBS, pH = 7.4, from freshly depolymerized paraformaldehyde as a fixative (*n* = 3 for each genotype). The tissue was removed with a sterile knife and subsequently transferred into ascending (10%, 20%, 30%) concentrations of sucrose at 4°C overnight. The material was then embedded in Tissue-Tek^®^ O.C.T. Compound (Sakura Finetek, Torrance, USA), cut into 8 μm thick sections in a cryostat (HM 500 OM, Microm, Walldorf, Germany), mounted on SuperFrost^®^ Plus adhesion slides (Menzel-Gläser, Braunschweig, Germany) and stored at −20°C. The frozen sections were moistened and then incubated with blocking buffer (PBS, pH = 7.4; containing 10% goat serum and 0.5% Triton X-100). GlyRα subunits were stained using mab4a (pan-GlyR-α) followed by a Cy3 conjugated goat anti-mouse IgG antibody (Jackson Immunoresearch Laboratories, West Grove, PA, USA). The glutamate receptor GluR1 was labeled by a polyclonal rabbit anti GluR1 antibody (BD Biosciences Pharmingen, San Diego, CA, USA) an Alexa Fluor^®^ 488 goat anti-rabbit IgG antibody (Invitrogen, Karlsruhe, Germany). In general, antibodies were diluted 1:100 in PBS containing 0.2% Triton X-100. After the addition of the primary antibody, the slides were kept at 4°C for 24 h. Washes (3 × 10 min in PBS) were performed after the incubation with the first and second antibodies. The secondary antibody was allowed to bind for 1 h at room temperature. Fluorescence pictures were acquired with a confocal microscope (TCS/SP2 DM-IRE2 workstation, Leica Microsystems, Bensheim, Germany) equipped with krypton-argon and helium-neon lasers. Quantification was performed as described previously (Groemer and Klingauf, 2007). In brief, sections were aligned on basis of GluR1 fluorescence, and the perpendicular was dropped to the edge of the dorsal horn. After cluster detection using a á-trous-wavelet transformation, the number of clusters were counted in 10 μm binnings from the dorsal edge of the dorsal horn over a length of 100 μm per section (*n* > 4 from at least three different animals per genotype). All evaluations were performed using custom written algorithms in IGOR-pro (Wavemetrics, Lake Oswego, OR, USA).

### Behavioral Assays

Mice were kept in groups (4–6 animals). A blinding of the experiments was not possible as phenotypes were easily identified. In case startle reactions occurred during or directly before the beginning of the measurements, the tests were aborted. In that case, animals were re-assessed on another day unless they had received an injection. Habituation was performed on the day before the measurement for rotarod, hotplate, and tailflick assays. For zymosan and formalin tests, mice were placed in the grid-cage or glass-terrarium 45 min before injection.

### Rotarod Motor Coordination Test

Motor performance was assessed by an automated rotarod test (Rota-Rod treadmill for mice 7600, TSE, Bad Homburg, Germany) at different velocities. The mice (*n* = 10 for each genotype) were allowed to habituate to the experimental setting for 3 min for each speed, with an unlimited number of trials on the rod. The mice were trained and tested using constant speeds of 18 rpm (28 cm/s), 24 rpm (38 cm/s), and 30 rpm (47 cm/s; diameter of the rod: 3 cm). On the day of the test, the mice were given three trials of a maximum of 60 s with a 60 s inter-trial interval for each speed. The mean time spent on the rod at a given speed was defined as running time.

### Hotplate/Tailflick Test

The response to noxious heat was tested with two different assays (*n* = 10 for each genotype). In a hotplate test, we used a heatable operating-table set to *T* = 53°C (Medax, Kiel, Germany). A plastic cylinder (diameter 16 cm, height 20 cm) was used to limit the range of motion. Habituation was performed on the plate at room temperature for 3 × 30 s for each mouse. Latency to the first pain-related behavior (PRB, i.e., flinching of a rear leg, paw licking, or jumping) was measured. Directly after this reaction, the mouse was removed from the setting to avoid tissue damage. Flinching or licking at the paw after completion of the test was not observed. On the day of the test, the mice were given three trials with an inter-trial interval >5 min and averages were defined as mean flinching latencies (MFL).

For the tail-flick test, an electronically controlled infrared Tail Flick Unit (Ugo Basile, Comerio, Varese, Italy) was used. During the measurements, mice were held in a plastic tube already present in their cages. The intensity of the infrared source was adjusted to produce a tailflick-latency (TFL) around 10 s. The time was taken automatically by the apparatus. The cut-off time was set to 20 s. As in the hotplate test, the mice were given three trials with an interval between the trials >5 min.

### Formalin Assay

Acute and delayed spontaneous pain-related behavior were measured in the formalin assay (Dubuisson and Dennis, 1977). Injection of formalin in the dorsal surface of the hind paw results in a reproducible biphasic pain-related behavior. The evoked PRB lasts for about one hour and is divided into an acute (phase 1) and delayed phase (phase 2) which are separated by a period of relative quiescence in PRB (interphase; Abbott et al., 1995). Phase 1 is thought to result from the direct activation of afferent fibers after the injection whereas phase 2 represents both tissue damage in the peripheral tissue and central sensitization. We have injected 20 μl of 2.5% formalin (in PBS, pH 7.4) subcutaneously into the back of the right hind paw with a GASTIGHT^®^ Hamilton-syringe (*n* = 8 for each genotype, formalin = 37% aqueous solution of formaldehyde; Cravatt et al., 2001). The duration of licking (s) and the number of flinches (n) on the injected paw were measured separately for 60 min for every minute immediately after the injection.

### Zymosan Assay

Mechanical sensitivity was determined before (baseline) and after zymosan-induced local inflammation using von-Frey filaments of defined strengths to evoke paw withdrawal and determine its threshold (withdrawal threshold, WTh; *n* = 6 for each genotype). Animals were placed on a platform with a wide gauge wire mesh bottom through which the hind paw could be stimulated. Observations were scaled ordinally attributing the values 0, 1, or 2 for “no visible reaction”, “visible reaction other than flinching”, and “flinching of the paw”, respectively. Three stimulations were performed per filament with an inter-stimulus-interval of more than 3 min and the WTh was defined as the first strength able to produce a mean value greater than 1.0 on the reaction scale. Left and right hind paws were measured in comparison for the determination of baseline-reproducibility. Inflammation was induced by injecting 20 μl Zymosan A solution (3 mg/ml Zymosan A from Sigma, Deisenhofen, Germany, in PBS, pH 7.4) subcutaneously into the plantar side of the left hind paw with a GASTIGHT^®^ Hamilton-syringe. WThs were measured before and each hour until 4 h after the injection.

### Statistical Analysis

Data were analyzed using Origin software (origin 7G, OriginLab Corporation, Northampton, USA). Significance was calculated using the Student’s t-test for unpaired samples. Asterisks indicate the level of significance *p < 0.05; **p < 0.01; ***p < 0.001. Error bars correspond to the S.E.M.

## Results

### Distribution of GlyR-Transcripts in Spinal Cord

**Figure 1:**
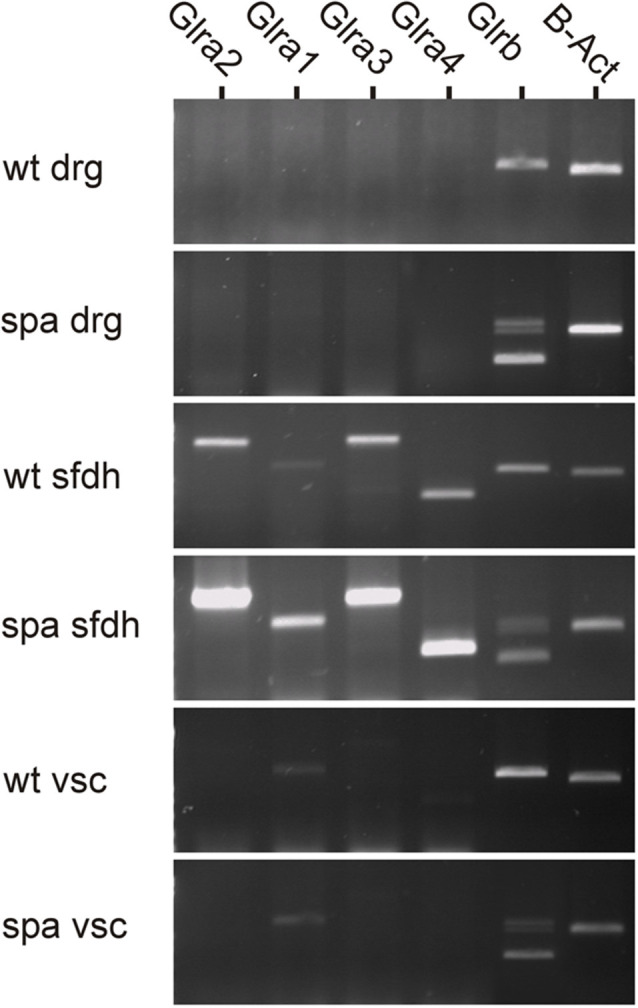
Region-specific expression of GlyR mRNA at the spinal level. GlyR mRNA expression was investigated in dorsal root ganglia (drg), superficial dorsal horn (sfdh) and ventral horn of the spinal cord (vsc). For GlyRβ, multiple bands in tissues derived from Glrb^spa/spa^ animals correspond to truncated transcripts generated by exon skipping (413 bp, transcript lacks exons 4 and 5; 643 bp, transcript lacks exon 5); the upper band represents the full-length transcript (726 bps; Mülhardt et al., 1994). No GlyRα subunit RNA could be detected in DRGs, whereas GlyRβ was expressed. Transcripts encoding GlyRα1–4 and GlyRβ were found in the superficial dorsal horn (sfdh; sizes: α1: 831 bp; α2: 1,152 bp; α3: 1,158 bp; α4: 509 bp; β - full length: 726 bp). The predominant subunit in the ventral spinal cord (vsc) was GlyRα1 (low signal). In Glrb^spa/spa^ mice, the regional distribution of glycine receptor mRNAs remained unchanged.

To analyze possible changes in the expression of the GlyR subunits in Glrb^spa/spa^ mice, RT-PCR analysis was performed of different regions at the spinal level (Figure 1). In samples from dorsal root ganglia, no GlyR α-subunits specific amplicons could be detected. Despite the lack of GlyRα subunit expression in this tissue, a single amplicon specific for GlyRβ subunit mRNA was detected in samples from wild-type animals. In contrast, multiple GlyRβ specific amplicons and only drastically reduced levels of amplicons corresponding full length GlyRβ were observed in dorsal root ganglion samples from spastic animals consistent with previous findings showing in addition to the correctly spliced full-length transcript represented by the upper band, truncated transcripts generated by exon skipping (Becker et al., 1992; Mülhardt et al., 1994). In samples from the spinal cord, additional transcripts specific amplicons for GlyRα subunits could be amplified. Here, α1 transcripts were found to be the prevailing α subunit in the ventral horn, whereas the expression levels of the other α subunits were below the detection level. In the sfdh, α2-α4 transcripts were present at higher levels as compared to α1 transcripts. Despite the reduction in the expression of the β subunit caused by the exon skipping observed in Glrb^spa/spa^ mice, the regional expression pattern of the α subunits was indistinguishable from that observed in wild-type animals.

### Glycine Receptor Immunofluorescence

**Figure 2:**
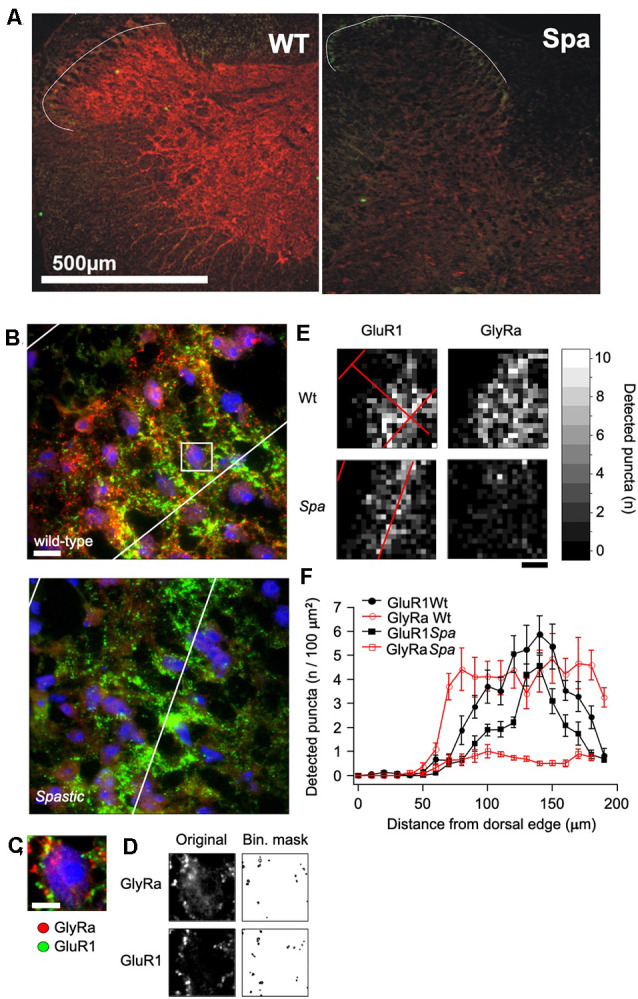
Distribution of GlyR immunoreactivity in the spinal cord. **(A)** Confocal images of glycine receptor α-subunits (GlyRa; mAb4a; red) and AMPA receptors (GluR1; green) immunoreactivity in spinal cord sections of mice with the indicated genotype. The white line indicates the outer boundary of GluR1 fluorescence, which marks the superficial layers (layer I-III) of the sfdh. **(B)** Example of an immunoflourescence picture of the GlyR and GluR1 fluorescence in the sfdh that was used for quantification (Scale bar = 20 μm). **(C)** Enlargement of the area within the presumptive layer II of the sfdh depicted in **(B)**. Note that all detectable immunofluorescence is localized in clusters. **(D)** Overview of GlyRα and GluR1 immunoreactive clusters detected by á-trous-wavelet transformation. **(E)** Spatial distribution of cluster densities in the confocal images presented in **(B)**. **(F)** Quantification of the cluster densities in relation to the distance from the surface of the sfdh given in 10 μm binnings.

To determine, if the reduced expression of full length GlyRβ affects the distribution of GlyRs at the protein level, immunohistochemical analysis of spinal cord sections of wildtype and spastic animals was performed (Figure 2). GlyRα subunit immunoreactivity (GlyRα-IR) was visualized by using the pan-GlyR α subunit antibody mAb4a. In sections from wild-type animals GlyRα-IR was observed to be localized in clusters throughout the gray matter of the spinal cord. As described previously (Puskár et al., 2001), GlyRα-IR cluster density was lower in the superficial layers of the dorsal horn (laminae I-III) as compared to other areas of the spinal cord, allowing at least in sections from wild-type animals a delineation of laminae I-III (Figures 2A,B). The alignment of the layering of different sections was verified by staining for the GluR1 subunit of the AMPA-type glutamate receptor, which is associated with inhibitory interneurons in lamina I-III, primarily in lamina II (Kerr et al., 1998). Clusters of GluR1 immunoreactivity (GluR1-IR) could be identified in lamina I-III with a focus on lamina II (Figures 2A,B). Some GluR1-IR was also detected in the deep dorsal horn (approx. lamina V). In individual sections, GluR1-IR clusters were found to produce an exclusion image with glycine receptor α subunit signal, reflecting the virtually complete separation of excitatory and inhibitory postsynaptic densities as described earlier (O’Brien et al., 1997). GluR1-IR was not detected in motoneurons in the ventral horn, where GlyRα-IR clusters were found on neuronal cell somata, dendrites, and axons (data not shown). In comparison to section from wild-type mice, GlyRα-IR was severely reduced in the spinal cord from spastic mice. There were, however, still some clusters of much lower staining intensity detectable that were not detectable with photomultiplier settings sufficient for wild-type analysis. Quantification revealed a highly significant reduction in the number of GlyR immunreactive clusters in all regions analyzed (Figures 2C–F). In addition, GluR1-IR staining localization was not changed (Figures 2A,B). Although GluR1 staining intensity and thereby also the number of detectable clusters appeared to be slightly reduced, these changes were not significant (Figure 2F).

### Motor Performance

**Figure 3:**
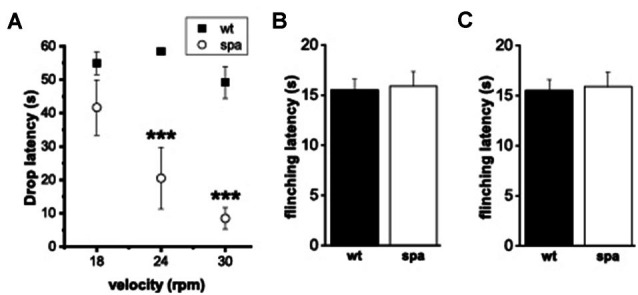
Motor performance and pain-related behavior. **(A)** Rotarod-test; mean time on the Rotarod. Motor ability was decreased markedly in mutant mice. **(B,C)** Heat-sensitivity assays. **(B)** Tail-flick assay; mean tail flick latencies. **(C)** Hotplate assay; mean reaction latencies. No differences were observed between the genotypes(***p < 0.001).

Although homozygous Glrb^spa/spa^ offspring derived from C57Bl/6J Glrb^+/spa^ × F1 (C57Bl/6J Glrb^+/spa^ × C3H/HeJGlrb^+/+^) matings reached adulthood without any increased mortality, they still showed apparently impaired motor coordination. Differences in the motor performance of spastic mice and wild-type littermates were assessed by an automated rotarod (*n* = 10 mice/genotype, Figure 3A). Here, the motor ability of mutant mice was significantly reduced, especially at higher velocities (at 18 rpm: 55 ± 3 s vs. 41 ± 8 s; at 24 rpm: 58 ± 1 s vs. 20 ± 9 s and at 30 rpm: 49 ± 5 vs, 8 ± 3 s for wt and spa respectively; *p* < 0.001, mean + SEM).

### Response to Acute Noxious Heat

To test if the deficits in synaptic glycinergic inhibition affect the reaction to acute pain elicited by thermal stimuli two different methods were used, the tail-flick- and the hotplate-assay (*n* = 10 mice/genotype; Figures 3B,C). In each test, the latencies of the behavioral response, tail-flick, and flinching, respectively, were measured. There were no significant differences between the two genotypes in the tail-flick-assay (wt: 10.9 ± 0.8 s vs. spa: 11.3 ± 1.1 s; Figure 3B). Equally, in the hotplate-assay, the mean flinching latencies were within the same range (wt: 15.5 ± 1.09 s vs. spa: 15.9 ± 1.43 s; Figure 3C). These findings are consistent with the hypothesis that the reaction to these painful stimuli was not affected by the motor impairment caused by the reduced GlyRβ expression observed in Glrb^spa/spa^ mice.

### Sensitivity to Noxious Chemical Stimuli (Formalin-Assay)

**Figure 4:**
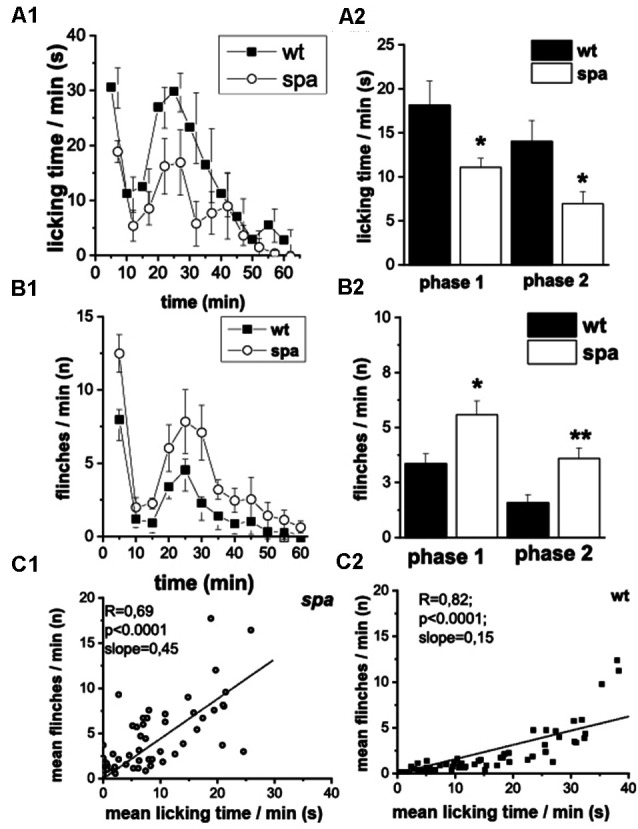
Formalin test; PRB after formalin injection. **(A)** Time course of licking activity (left panel; **A1**) and mean licking time in phase 1 (0–10 min) and 2 (right panel; **A2**). The licking time was reduced in spastic animals during the 60 min of the test. **(B)** Time course of flinches **(B1)** and mean flinching in phase 1 and 2 **(B2)**. The number of flinches per minute was increased in mutant mice in both phases. Phases of both acute (0–15 min, phase 1) and delayed (>15 min phase 2) PRB could be clearly distinguished by measuring either flinching or licking-time. **(C)** Mean flinching frequency over the mean licking time frequency at individual minutes comparing spastic **(C1)** and wildtype **(C2)** mice. The number of flinches per minute was correlated positively with the mean licking time (*p < 0.05; **p < 0.01).

Subsequently, the pain-related behavior (PRB) in response to a chemical stimulus was assessed, that in addition to activating pain transducing afferent fibers also is suggested to cause central sensitization. Here, after injection of 2.5% formalin into the right hindpaw (*n* = 8 animals/genotype), the number of flinching and licking responses of the animals over a period of 60 min starting immediately after the injection were scored. Compared to their wild-type littermates, spastic mice exhibited significant changes in the reaction in both the acute (0–15 min; phase 1, p1) and the delayed phase (>15 min; phase 2, p2) of the test (Figures 4A1,B1). Whereas a decrease in licking time/min was observed in both phases in mutant mice (p1: wt: 18.1 ± 2.8 s vs. spa: 11.1 ± 1.0 s, *p* < 0.05; p2: wt: 14.0 ± 2.3 s vs. spa: 6.9 ± 1.4 s, *p* < 0.05; Figure 4A2), the number of flinches/min was significantly increased in both phases (p1: wt: 3.4 ± 0.4 vs. spa: 5.6 ± 0.6, *p* < 0.05; p2: wt: 1.6 ± 0.4 vs. spa: 3.6 ± 0.5, *p* < 0.01; Figure 4B2). Control injections of PBS did not evoke any PRB. Since both liking and flinching are supposedly pain elicited behavioral responses, we tested whether the licking and flinching frequency correlate. A good correlation would suggest that both values are predominantly dependent on the same variable, i.e., most likely the pain intensity. This hypothesis was tested by a regression analysis. Despite the diverging effect of the Glrb^spa^ mutation on the licking and flinching frequency, the mean number of flinches/min correlated well with the mean time spent licking/min for both mutant and wildtype mice (Figures 4C1,C2; spa: *n* = 60; *R* = 0.69, *p* < 0.001; wt: *n* = 60; *R* = 0.82, *p* < 0.001).

### Sensitivity to Mechanical Stimuli in Native and Inflamed Tissue (Zymosan-Assay)

**Figure 5:**
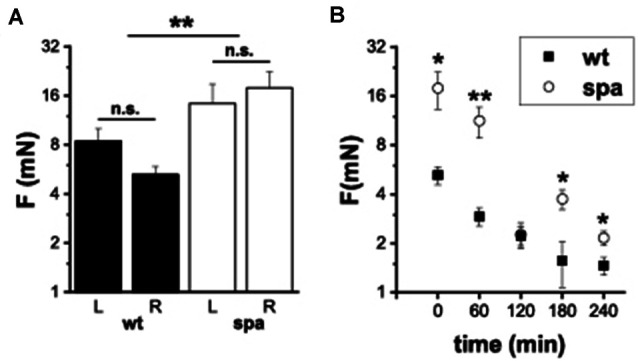
Mechanically induced nocifensive behavior after zymosan A injection into the right hind paw. **(A)** Baseline reproducibility; mean withdrawal thresholds (L, left paw; R, right paw); there were no significant differences between the withdrawal thresholds of left and right paw subgroups. WTh’s were elevated in the spastic group. **(B)** Time course of mean WTh’s after zymosane A injection; both, spastic and wildtype mice develop allodynia. *p < 0.05; **p < 0.01; n.s., not significant. Error bars indicating S.E.M.

It was shown previously that defects in glycine-dependent neurotransmission like the loss of the GlyR subunit GlyRα 3 resulted in changes in inflammatory pain. To investigate if the reduced expression of the β subunit of the GlyR is also associated with an impaired development of inflammatory pain, the changes in mechanical sensitivity after injection of Zymosan A into the right hindpaw of wildtype and spastic mice were analyzed. The paw withdrawal thresholds (WThs) were determined using von-Frey filaments (*n* = 6 mice/genotype; Figure 5). Although there were no significant differences between the withdrawal thresholds of left and right paw subgroups before Zymosan A injection in both subgroups (wt left: 8.4 ± 1.6 mN; wt right: 5.2 ± 0.6 mN; spa left: 14.3 ± 4.5 mN; spa right: 17.9 ± 4.6 mN), the WThs were elevated in the spastic group (wt: 6.8 ± 1.0 mN; spa: 16.1 ± 3.1 mN; *p* < 0.01). After injection of Zymosan A, however, an inflammation induced allodynia as indicated by a significant reduction in the WTh of the right paw was observed for both genotypes (Figure 5B; 1 h after injection: wt: 2.9 ± 0.4 mN vs. spa: 11.2 ± 2.4 mN, *p* < 0.01; 2 h after injection: wt: 2.2 ± 0.3 mN vs. spa: 2.3 ± 0.4 mN, *p* > 0.1; 3 h after injection: wt: 1.6 ± 0.5 mN vs. spa: 3.8 ± 0.5 mN, *p* < 0.05; 4 h after injection: wt: 1.5 ± 0.2 mN vs. spa: 2.2 ± 0.2 mN, *p* < 0.05).

## Discussion

It was demonstrated previously that the LINE1 element insertion into the intronic region before exon 6 of the *Glrb* gene causes missplicing of the *Glrb* hnRNA by exon skipping in a major fraction of the mature Glrb mRNA, although some correctly spliced mRNA can still be found (Mülhardt et al., 1994; Becker et al., 2012). Consistent with these findings, we found in this study that misspliced Glrb mRNA can be detected throughout the spinal region including the dorsal root ganglion. Consistently, full length Glrb transcripts were detectable, in preparations form wild-type animals. Here, no GlyRα specific amplicons could be detected in this tissue, thus contrasting previous studies that have shown GlyRα protein in this tissue (Wang et al., 2018; Yao et al., 2021). Whether the protein detected here is localized on spinal projections, or whether the too low sensitivity of our assay system precluded a detection, however, is unclear at present. In the sfdh, amplicon specific for all four GlyRα isoforms are detected. Here, they might be expressed by distinct neuronal populations and serve diverging functions. Recently, PGE2 induced PKA-dependent phosphorylation of the GlyRα3 subunit has been demonstrated in the process of inflammatory pain sensitization at the spinal level (Harvey et al., 2004; Werynska et al., 2021). In contrast to the sfdh, only GlyRα1 and GlyRβ transcripts could be detected in the ventral spinal cord. This is consistent with the observation that mutations affecting GlyRα1 and β subunits result in a startle phenotype (Becker et al., 1986), whereas GlyRα3 knockout mice do not show any motor impairment (Harvey et al., 2004). Although the spatial expression pattern of GlyR subunit mRNA was found in spastic mice was similar to that found in wild-type controls, only the expression of the full length GlyRβ was found to be significantly reduced, whereas the expression level of the GlyRα subunit was maintained if not enhanced when compared to control samples as assessed by semiquantitative PCR. In contrast, our immunohistochemical data demonstrate a significant reduction in staining intensity throughout the spinal cord, demonstrating that the spastic mutation results in a reduced glycine receptor specific immunofluorescence in the whole spinal cord of the C57Bl6/C3H hybrids analyzed here. Previous studies have demonstrated that the used antibodies recognize preferentially clustered GlyRs, whereas diffusely GlyRs dispersed in the plasma membrane are only insufficiently labeled. Thereby these findings confirm that the reduction in functional β subunits expression results in a reduced synaptic clustering mediated by gephyrin—GlyRβ interaction, thus causing fewer GlyR clusters albeit no reduction in transcript levels are detectable.

It has been shown previously, that the reduced number of postsynaptic GlyRs at inhibitory synapses of Glrb^spa/spa^ mice causes significant changes in glycinergic input on motoneurons (von Wegerer et al., 2003). This has been suggested to be causal for the motor phenotype observed in Glrb^spa/spa^ mice. Thus, the motor phenotype of the spastic mutant might also compromise the pain associated behavioral responses that require the animal to move. Our data show that in Glrb^spa/spa^ mice motor reactions, however, are not uniformly shifted (to a hyperreactivity) but changed depending on the sensory input, i.e., unchanged responses to heat, enhanced flinches in the formalin test, and reduced reactions upon mechanical stimuli. Although we cannot exclude some influence of the impaired motoperformance on the results obtained here, the observed changes in pain associated behavior cannot be explained solely by alteration in the excitability of motoneurons alone.

The heat-induced tail flick is a spinal reflex that can be evoked even in decerebrate animals (Matthies and Franklin, 1992) demonstrating that this behavioral response is mediated exclusively by neuronal circuitries within the spinal cord. If disinhibition in Glrb^spa/spa^ mice affects exclusively the output or motor side, one would expect a facilitation of movement and hence lower thresholds. Moreover, if disinhibition also occurred on the sensory side, effects should be additive. In *spastic* mice, however, we observed no differences in tail-flick latencies compared to wild-type animals.

Chemically evoked (i.e., formalin-induced) pain-related behavior resulted in enhanced flinching and reduced licking times in the mutants. When observed over the time course of the test, the mean number of flinches/min was correlated positively with the mean time spent licking/min in both wild-type and mutant mice. Thus, the Glrb^spa/spa^ mice did not show more flinching at the cost of licking in mutants. Moreover, as these parameters are affected in opposite directions in spastic mice, we consider it relevant to separate those two parameters from each other, as they are easily distinguishable and can be measured simultaneously. Flinching is assumed to represent a direct reaction to incoming nociceptive signals that are processed spinally and promote the escape from potential tissue damage (Coderre et al., 1994). The unchanged flinching reaction in the hotplate test demonstrated that the primary neurocircuit mediating this escape response is intact in Glrb^spa/spa^ mice at large. The enhanced reactions in the formalin test, however, suggest differences in the input modalities or alternatively in the intraspinal processing, like, e.g., central sensitization.

In contrast to the flinching response, the licking at the paw requires supraspinal input (Weijnen, 1998) and may represent an alleviating behavioral component making use of spinal segmental gate control mechanisms. To this end, tactile stimulation can reduce nociceptive input *via* synaptic glycinergic inhibition (Narikawa et al., 2000). The decrease in licking time in spastic mice could therefore be caused by chronic disinhibition in the dorsal horn resulting in reduced efficacy of gate control mechanisms and separation of modalities. As a consequence, spastic mice might experience less benefit from licking as a lateral inhibition maneuver.

Previous studies have shown that intrathecal application of the GlyR antagonist strychnine evokes allodynia (Yaksh and Harty, 1988). This is thought to reflect the spinal disruption of glycinergic inhibition which causes activation of low-threshold mechanoreceptive afferent inputs, inducing nocifensive behavior upon otherwise innocuous stimuli (Sherman et al., 1997). Similarly, the acute reduction of glycinergic inhibition by loss of the glycinergic neurons itself was shown to result in enhanced spontaneous itching and/or pain response (Foster et al., 2015). This contrasts with our findings in Glrb^spa/spa^ mice, that despite chronic glycinergic disinhibition an increased withdrawal thresholds to light mechanical but not heat stimuli was observed. These findings suggest that spinal sensory discrimination (noxious/innocuous) is influenced differentially by the mutation. Electrophysiological recordings from neurons in the nucleus hypoglossus (Muller et al., 2008) and from the sfdh of homozygous spastic animals revealed a compensatory increase in GABAergic currents that could not be detected in the ventral horn (von Wegerer et al., 2003); this could allow for a normal processing of heat stimuli while the transmission of tactile input may be reduced. Accordingly, our data suggest that the disruption of spinal sensory contrast enhancement in spastic mice affects predominantly reactions upon low threshold inputs (i.e., mechanical thresholds/formalin-induced licking) whereas reactions upon high threshold inputs (i.e., formalin-induced flinching, noxious heat thresholds) are unaltered or enhanced. Future studies will have to address the region and cell type specific composition of GlyRs as well as the specific functions of synaptic glycinergic inhibition on sensory information processing, and possible compensatory mechanisms in case of chronic deficiency in synaptic inhibition.

## Data Availability Statement

The raw data supporting the conclusions of this article will be made available by the authors, without undue reservation.

## Ethics Statement

The animal study was reviewed and approved by Regierung von Unterfranken.

## Author Contributions

TG and KB performed the experiments described in this article. TG, KB, VE, and CMB were involved in organizing the data and wrote the manuscript. TG, HUZ, AT, KB, and CMB designed the experiments. All authors contributed to the article and approved the submitted version.

## Conflict of Interest

VE works as a consultant for Disc medicine, this was, however, completely independent of the work presented here. The remaining authors declare that the research was conducted in the absence of any commercial or financial relationships that could be construed as a potential conflict of interest.

## Publisher’s Note

All claims expressed in this article are solely those of the authors and do not necessarily represent those of their affiliated organizations, or those of the publisher, the editors and the reviewers. Any product that may be evaluated in this article, or claim that may be made by its manufacturer, is not guaranteed or endorsed by the publisher.

## Abbreviations

PRB: pain-related behaviour
PCR: polymerase chain reaction
RPM: rounds per minute
spa: spastic
sfdh: superficial dorsal horn
TFL: tail flick latency
wt: wildtype
WTh: withdrawal threshold(s).
